# Phospholipase D2 is a positive regulator of sirtuin 1 and modulates p53-mediated apoptosis via sirtuin 1

**DOI:** 10.1038/s12276-021-00659-y

**Published:** 2021-09-01

**Authors:** Hyesung Lee, Taek-Yeol Jung, Seong Hun Lim, Eun Ju Choi, Jinu Lee, Do Sik Min

**Affiliations:** 1 College of Pharmacy, Yonsei University, Incheon, 21983 South Korea; 2 Department of Life Science, College of Natural Science, Ewha Womans University, Seoul, 03760 South Korea; 3 Yonsei Institute of Pharmaceutical Sciences, Yonsei University, Incheon, 21983 South Korea

**Keywords:** Lipid signalling, Cancer metabolism

## Abstract

Sirtuin 1 (SIRT1) is a nicotinamide adenine dinucleotide-dependent histone deacetylase that plays diverse physiological roles. However, little is known about the regulation of SIRT1 activity. Here, we show that phospholipase D2 (PLD2), but not PLD1, selectively interacts with SIRT1 and increases the deacetylase activity of SIRT1. PLD2 does not interact with the other isozymes of SIRT (SIRT2–7). Two leucine residues in the LXXLL motif (L173 and L174) in the phox domain of PLD2 interact with the region essential for SIRT1 activity. PLD2 stimulates the SIRT1-mediated deacetylation of p53 independent of its lipase activity. In our study, mutagenesis of the LXXLL motif suppressed the interaction of PLD2 with SIRT1 and inhibited SIRT1-mediated p53 deacetylation and p53-induced transactivation of proapoptotic genes. Ultimately, overexpression of wild-type PLD2 but not that of LXXLL-mutant PLD2 protected cells against etoposide-induced apoptosis. Moreover, PLD2 did not protect against apoptosis induced by SIRT1 depletion under genotoxic stress. Collectively, our results suggest that PLD2 is a positive regulator of SIRT1 and modulates p53-mediated apoptosis via SIRT1.

## Cancer: Regulating tumor cell self-defense

New details about the regulatory mechanisms that prevent tumor cell death could be exploited to increase the effectiveness of chemotherapy. The sirtuin (SIRT) protein family has been linked to both promotion and suppression of tumors in different cancers. The enzyme SIRT1 in particular deacetylates, and thereby deactivates, the key tumor-suppressing antigen p53, stopping p53 from inducing apoptosis (controlled cell death) in tumors. Do Sik Min at Yonsei University, Incheon, South Korea, and co-workers revealed that this SIRT1 deacetylation of p53 is greatly enhanced by the activity of the enzyme phospholipase D2 (PLD2). A particular region on PLD2 is required to activate SIRT1, this activation leading to protection of tumor cells from apoptosis induced by the chemotherapy drug etoposide. Therapies that target that region on PLD2 might therefore suppress a tumor’s natural resistance to chemotherapy.

## Introduction

Sirtuins (SIRTs) belong to a family of class III histone deacetylases (HDACs) that is composed of seven mammalian members of SIRTs (SIRT1–7) with a conserved catalytic core for which nicotinamide adenine dinucleotide (NAD^+^) is a cofactor^1–3^. Sirt1 regulates a wide range of cellular functions, including cell survival, senescence, aging, differentiation, and metabolism, through its deacetylation activity targeting numerous transcription factors. These transcription factors include p53, forkhead box O3 (FoxO3), nuclear factor-κB, and peroxisome proliferator‐activated receptor gamma coactivator‐1α^4^. Sirt1 activity is regulated by various endogenous factors. The deacetylase activity of Sirt1 is limited by cellular NAD^+^ levels, which fluctuate in response to cellular energy requirements and changing rates of NAD^+^ biosynthesis and consumption^5^. Sirt1 activity is also regulated by direct interactions with several proteins, including the positive regulators active regulator of Sirt1^6^ and Necdin^7^ and the inhibitory protein DBC1 (deleted in breast cancer)^8,9^. However, the molecular mechanisms that govern substrate selection by SIRT1 and the regulation of its deacetylation activity remain poorly defined. Both positive and negative regulations of Sirt1 activity have therapeutic implications against some diseases, including aging, diabetes, and tumors^10^. The SIRT protein family is important because of its seemingly dichotomous role in cancer biology, with both tumor promoter and tumor suppressor functions in different cancers. Identifying new regulators of SIRT1 activity would be helpful in understanding the regulatory network of SIRT1 and its biological relevance in cancer development and tumor therapeutics.

The identification of p53 as a bona fide substrate of Sirt1 deacetylation has linked Sirt1 to tumorigenesis. Sirt1 interacts with and deacetylates p53, thereby negatively regulating p53-mediated transactivation^11^. p53 acetylation promotes the transactivation of many specific genes, thus regulating cell cycle arrest, apoptosis, and metabolic targets. This enzymatic reaction is key to p53 regulation in tumor suppression. When p53 is not acetylated, MDM2 ubiquitinates p53, targeting it for degradation, thus suppressing p53 function^12^.

Phospholipase D (PLD) generates the lipid second messenger phosphatidic acid (PA), and it is abundant, overactivated, or is mutated in various human cancers. Furthermore, its activity in cancer cells has been linked to proliferative signaling, evasion of growth suppressors, resistance to cell death, and increased cell invasiveness and metastasis^13–15^. The PLD isoforms PLD1 and PLD2 have been linked to the prometastatic phenotype, although they likely have different functions in this context^16^. Inhibition of PLD activity is critical for an increase in p53 stability and suppression of MDM2 levels^17^. We have previously reported that PLD1 plays an antiapoptotic role, probably through the modulation of the p53-mediated cell death pathway^18^. The LXXLL motif (L is a leucine residue and X is any amino acid) was originally observed in a variety of coactivators and is described as a signature motif that mediates the recruitment of coactivators through the action of nuclear hormone receptors^19,20^. LXXLL motifs exhibit specific interaction profiles with their partner proteins and regulate different biological functions in a subtle manner via specific interactions^21^. Moreover, the LXXLL motif plays a critical role in the interaction of FoxO1 with Sirt1^22^.

In this study, we identified the presence of an LXXLL motif in PLD2 and demonstrated that the LXXLL motif (amino acids 170–174) of PLD2 plays a critical role in the selective interaction with and activation of Sirt1. Furthermore, we found that the transcriptional activity of p53 is dependent on the LXXLL motif of PLD2. PLD2 overexpression protected against etoposide-induced apoptosis via the Sirt1-mediated downregulation of proapoptotic genes. The PLD2 mutant in which two leucine residues at amino acids 173 and 174 are replaced by alanine did not protect against etoposide-induced apoptosis. In summary, we suggest that the LXXLL motif of PLD2 is required for positive SIRT1 regulation and may be a target site for Sirt1-mediated regulation of apoptosis.

## Materials and methods

### Cell culture and transfection

HEK293, A549, HCT116, and HT29 cells were maintained in Dulbecco’s modified Eagle’s medium with 10% fetal bovine serum and incubated at 37 °C in a humidified atmosphere of 5% CO_2_. The cells were transfected using Lipofectamine 3000 (Invitrogen, Carlsbad, CA, USA) according to the manufacturer’s instructions. Etoposide was purchased from Alexis (Lausen, Germany). Trichostatin A (TSA) was purchased from Cell Signaling Technology (Danvers, MA, USA). All other chemicals were purchased from Cayman Chemical (Ann Arbor, MI, USA) and Sigma Aldrich (St. Louis, MO, USA).

### Site-directed mutagenesis

Wild-type (wt) hPLD2 cloned into the pEGFP C1 plasmid served as a template vector for site-directed mutagenesis. Site-directed mutagenesis of the LXXLL motif in PLD2 was generated by polymerase chain reaction using a QuikChange site-directed mutagenesis kit (Stratagene, San Diego, CA, USA) according to the manufacturer’s instructions. The mutations were confirmed by DNA sequencing. The primers for generation of LXXLL mutant PLD2 were as follows: LXXAA123 (5′-forward, AGACACAAAGTCTTGATGAGTGCGGCCCCTCTGGCTCGA, and 3′-reverse, TCTGTGTTTCAGAACTACTCACGCCGGGGAGACCGAGCT) and LXXAA 173 (5′-forward, CTGGAGA ATTACCTCAACTGTGCCGCGACCATGTCTTTC, and 3′-Reverse, GACCTCTTAATGGAGTTGACACGGCGCTGGTACAGAAAG). Mutagenesis for the generation of LXXAA123/173 was performed using LXXAA123 as a template. This mutation was confirmed using DNA sequencing.

### Immunoprecipitation and western blot analysis

The following antibodies were used: anti-SIRT1, anti-p53, anti-BAX, anti-GFP, anti-Myc, anti-HA, anti-Flag (Santa Cruz Biotechnology, Dallas, TX, USA), and anti-acetyl-p53 (K382) (Cell Signaling Technology). A polyclonal antibody that recognizes both PLD1 and PLD2 was generated as previously described^22^. Signal densities of the blots were measured with ImageJ software (Wayne Rasband, NIH, Bethesda, MD, USA) and normalized using an anti-α-tubulin antibody.

### Luciferase reporter assays

HEK293 cells were transiently transfected with *Bax*- or *Noxa-*luciferase reporter genes using Lipofectamine 3000 reagent (Invitrogen). Luciferase activity was measured using the dual-luciferase reporter assay system (Promega, Madison, WI, USA) according to the manufacturer’s instructions. Relative luciferase activity was obtained by normalizing firefly luciferase activity to *Renilla* luciferase activity.

### Glutathione-S-transferase (GST) pull-down assay

*Escherichia coli* BL21 cells were transformed with individual expression vectors encoding GST fusion proteins. The generated GST fusion proteins were purified using glutathione sepharose 4B. The cell lysates were incubated with GST fusion proteins immobilized on glutathione sepharose 4B beads for 1 h at 4 °C. The bound proteins were analyzed by immunoblotting.

### Caspase-3 activity

Caspase-3 activity assay is based on the ability of caspase-3 to hydrolyze a specific substrate linked to the fluorescent probe rhodamine 110. Under these conditions, the substrate linked to rhodamine 110 is nonfluorescent (Z-DEVD-rhodamine 110). Cleavage of the caspase substrate from the probe leads to the generation of a measurable fluorescent signal. The protocol for the assay was obtained from a EnzChek caspase-3 fluorescent assay kit (Thermo Fisher Scientific, Waltham, MA, USA) and modified for use in six-well tissue culture plates using a microplate fluorometer (Wallac, Ramsey, MN, USA). After the treatment period, the medium in each well of the tissue culture plate was removed, and 50 μL of lysis buffer was added to each well. After 30 min of incubation on ice, 50 μL 2× reaction buffer containing 10 mM dithiothreitol and the caspase-3 substrate Z-DEVD-AMC (5 μM final concentration) was added to each well. Caspase-3 activity was monitored by measuring the fluorescence in each well with the fluorescent detector set to 342 nm excitation and 441 nm emission.

### In vitro translation

Direct interactions between SIRT1 and PLD2 were analyzed by immunoprecipitation using in vitro translation. SIRT1 and PLD2 were purified using a TnT T7 quick coupled transcription/translation reaction kit (Promega). Briefly, 0.5 μg of plasmid DNA containing SIRT1 or PLD2 was mixed with 40 μL of TnT master mix and 2 μL of methionine for 90 min at 30 °C. The in vitro translated proteins were mixed and incubated for 1 h. The mixture was then analyzed by immunoprecipitation and immunoblotting.

### In vitro deacetylation assay

The in vitro deacetylase activity of the SIRT1 assay was performed using a Flour de Lys-SIRT1 assay kit (Enzo Biochem, Farmingdale, NY, USA). The reactions were performed using in vitro translated wtPLD2, mtPLD2, or recombinant human SIRT1. Fifty microliters of reaction mixture contained 20 μM Fluor de Lys-SIRT1 substrate and 100 μM NAD^+^. After 10 min of incubation at 37 °C, the reaction was stopped by adding a fluorophore developer. The fluorophore was excited with 360 nm light and detected at 460 nm on a fluorometric plate reader. The p53 was acetylated in vitro as described previously^23^. The recombinant p300 histone acetyltransferase (HAT) domain (1 μg) was incubated with 3 μg GST-p53 in the presence of 70 μM acetyl-CoA. After p53 acetylation was verified by immunoblotting, the acetylated p53 protein was subjected to a deacetylation assay. One hundred nanograms of acetylated p53 was incubated with PLD2 (100 ng) in the absence or presence of 50 ng SIRT1. The level of p53 acetylation was examined by immunoblotting using antibodies against acetylated p53.

### Apoptosis assay using flow cytometry

Annexin V binding was assessed by flow cytometry using a commercial kit (Abcam, Cambridge, England) according to the manufacturer’s instructions. Cells were collected by centrifugation and resuspended in 500 μL of 1X annexin V binding buffer. Annexin V-FITC was added to the resuspended cells and incubated at room temperature for 5 min in the dark. Stained cells were analyzed by flow cytometry.

### Statistical analysis

The results are expressed as the mean ± standard deviation (SD) of the number of determinations indicated. The statistical significance of differences was determined using analysis of variance. *P* values < 0.05 or 0.01 were considered statistically significant.

## Results

### The LXXLL motifs are conserved among PLD2 isozymes, and PLD2 but not PLD1 selectively interacts with SIRT1

**Fig. 1:**
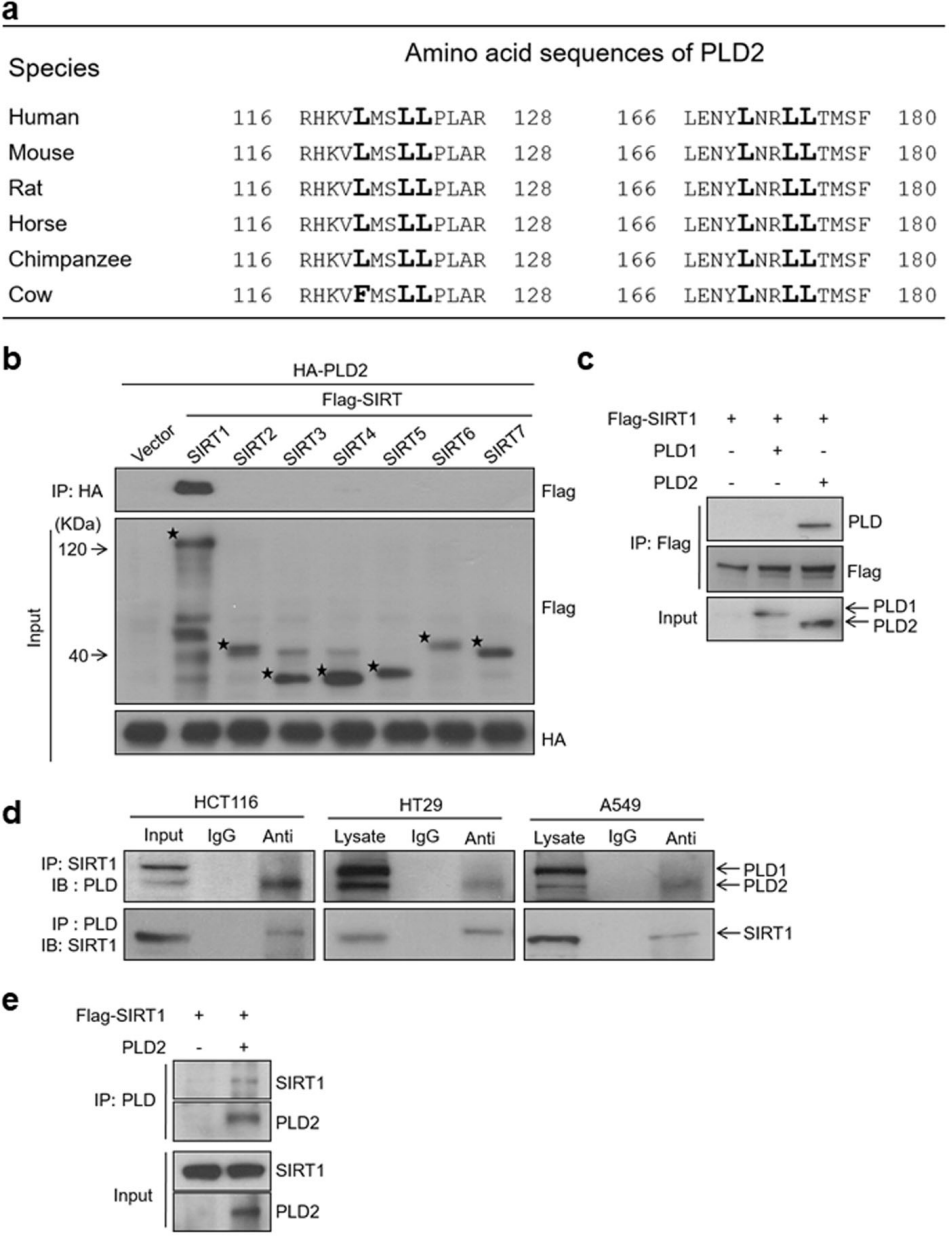
The LXXLL motifs are conserved among PLD2 isozymes, and PLD2 but not PLD1 selectively interacts with SIRT1. **a** Sequence alignment of LXXLL motifs in different species of PLD2. **b** HEK293 cells were cotransfected with PLD2 and the indicated SIRT constructs. The lysates were immunoprecipitated and/or immunoblotted using the indicated antibodies. **c** HEK293 cells were cotransfected with the indicated constructs, and the lysates were analyzed by immunoprecipitation and immunoblotting. **d** Endogenous interaction between SIRT1 and PLD2 in the indicated cells. **e** In vitro interaction of SIRT1 with PLD2. In vitro translated proteins were used to examine direct interactions, as analyzed by immunoprecipitation and immunoblotting. The results are representative of at least three independent experiments.

Since the LXXLL motif is involved in the interaction with PLD partner proteins, such as Sirt1^22^, we examined whether PLD isozymes contain LXXLL motifs. We identified two LXXLL motifs at amino acids 120–124 and 170–174 in human PLD2, which are highly conserved among PLD2 in mice, horses, chimpanzees, and cattle (Fig. 1a). However, LXXLL motifs were not present in PLD1. This finding suggests that the LXXLL motif may play a role in the selective function of PLD2. To verify the interactions between PLD2 and various Sirt families, SIRT1 deletion constructs were cotransfected, followed by immunoprecipitation and immunoblotting. Interestingly, PLD2 selectively interacted with only SIRT1 but not with SIRT2, 3, 4, 5, 6, or 7 (Fig. 1b). However, exogenous SIRT1 interacted with PLD2 but not PLD1, as analyzed by immunoprecipitation using an anti-PLD antibody that recognizes both PLD1 and PLD2 (Fig. 1c). To further confirm whether endogenous SIRT1 and PLD isozymes can form a natural complex in colorectal cancer cells (HT119 and HT29 cells) and A549 lung adenocarcinoma cells, which express both endogenous PLD1 and PLD2 (Fig. 1d), we performed coimmunoprecipitation assays. Both SIRT1 and PLD2, but not PLD1, were detected in immunoprecipitates captured by anti-SIRT1 and anti-PLD antibodies, while neither of these PLD isozymes were identified in the immune complexes formed with nonimmune IgG (Fig. 1d). These findings indicate a specific association between endogenous SIRT1 and PLD2. To further verify that the interaction between PLD2 and SIRT1 was direct, we conducted an in vitro binding assay using in vitro translated SIRT1 and PLD2 and found that PLD2 directly interacts with SIRT1 (Fig. 1e). Collectively, these results show that PLD2, but not PLD1, selectively interacts with SIRT1.

### The LXXLL motif of PLD2 interacts with the C-terminal region essential for SIRT1 activity

**Fig. 2:**
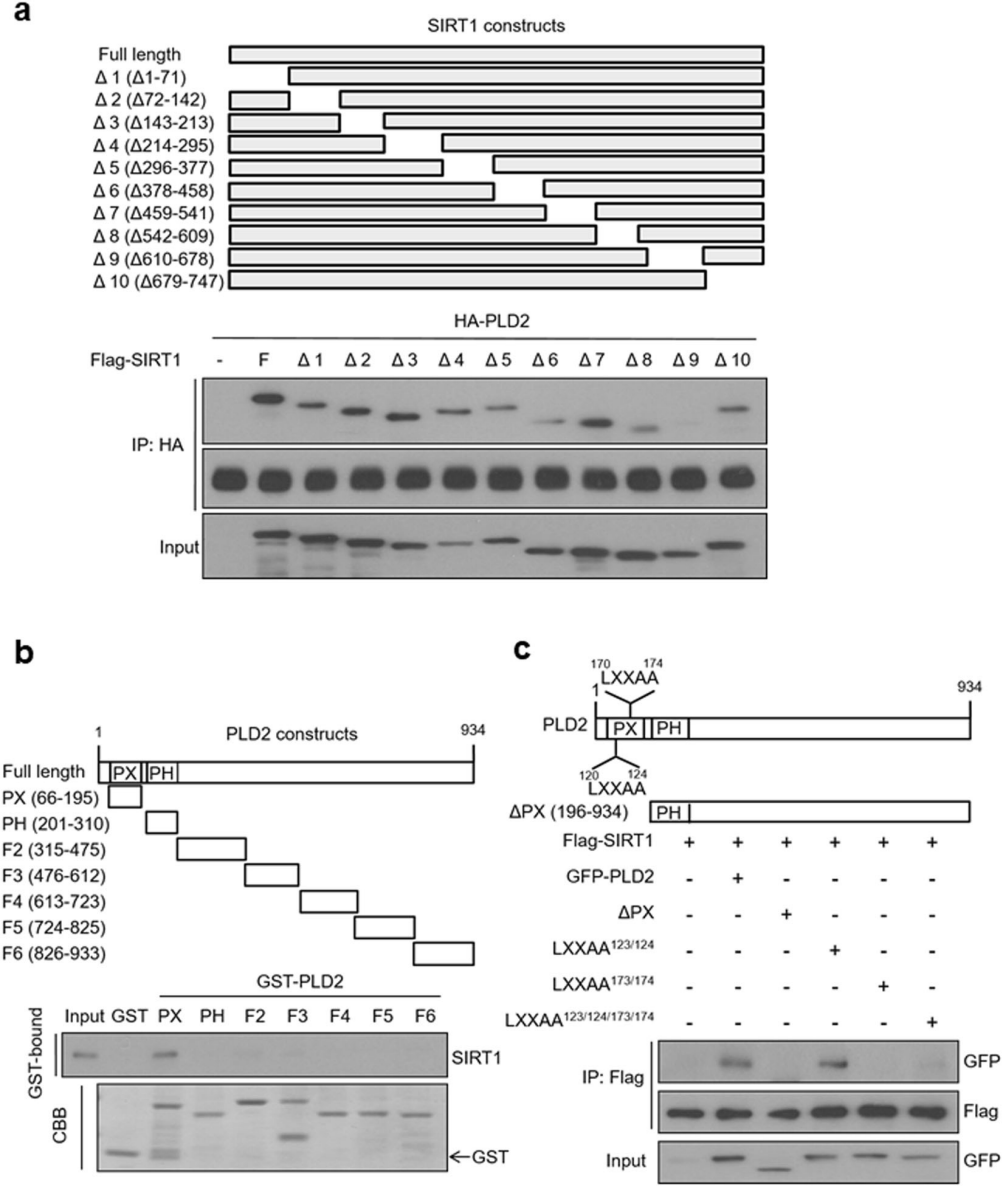
The LXXLL motif of PLD2 interacts with the C-terminal region essential for SIRT1 activity. **a** Schematic representation of various deletion mutant SIRT1 constructs. HEK293 cells were transfected with PLD2 and SIRT1 deletion mutants, and the lysates were analyzed by immunoprecipitation and immunoblotting using the indicated antibodies. **b** Schematic representation of various GST-PLD2 fragment fusion constructs. GST pull-down assays using equal amounts of GST or GST-PLD2 fragment fusion proteins immobilized on glutathione sepharose 4B beads were performed with HCT116 cell lysates and immunoblotting was performed with antibodies against SIRT1. The amount of GST fusion proteins was visualized by Coomassie brilliant blue (CBB) staining. **c** Schematic representation of LXXAA mutants of PLD2 and PX domain-deleted PLD2 (ΔPX). HEK293 cells were cotransfected with SIRT1 and the indicated PLD2 constructs and analyzed by immunoprecipitation and immunoblotting. The results are representative of at least three independent experiments.

To identify the regions of SIRT1 critical for the interaction with PLD2, various SIRT1 deletion mutants of were used for an immunoprecipitation assay (Fig. 2a). Deletion analysis showed that amino acids 610–678 of SIRT1 (SIRT1Δ9) are required for the interaction between SIRT1 and PLD2, since deletion of this SIRT1 region abolished the binding of PLD2 to SIRT1 (Fig. 2a). It was reported that amino acids 631–655 in the C-terminal domain are essential for SIRT1 deacetylase activity realized through its C-terminal^23^. To verify that these regions of PLD2 mediate the interaction with SIRT1, we conducted a GST pull-down assay using a panel of GST-PLD2 fragments. The results revealed that SIRT1 bound to the phox (PX) domain (66–195 amino acids) of PLD2 (Fig. 2b). Moreover, deletion mutants of the PX domain failed to interact with SIRT1 (Fig. 2c). The PX domain of PLD2 contains two LXXLL motifs. To investigate whether the LXXLL motifs of PLD2 are involved in the PLD2 association with SIRT1, we constructed LXXAA-mutant PLD2 in which leucine 123/124 and 173/174 were replace with alanine. The mutation of leucine 123 and 124 (LXXAA^123/124^) of PLD2 did not affect PLD2 interaction with SIRT1, but the LXXAA^173/174^-mutant PLD2 failed to associate with SIRT1 (Fig. 2c). These results indicate that leucine 173 and 174 in the LXXLL motif of PLD2 are required for PLD2 association with the C-terminus of SIRT1.

### PLD2 cooperates with SIRT1 to deacetylate p53

**Fig. 3:**
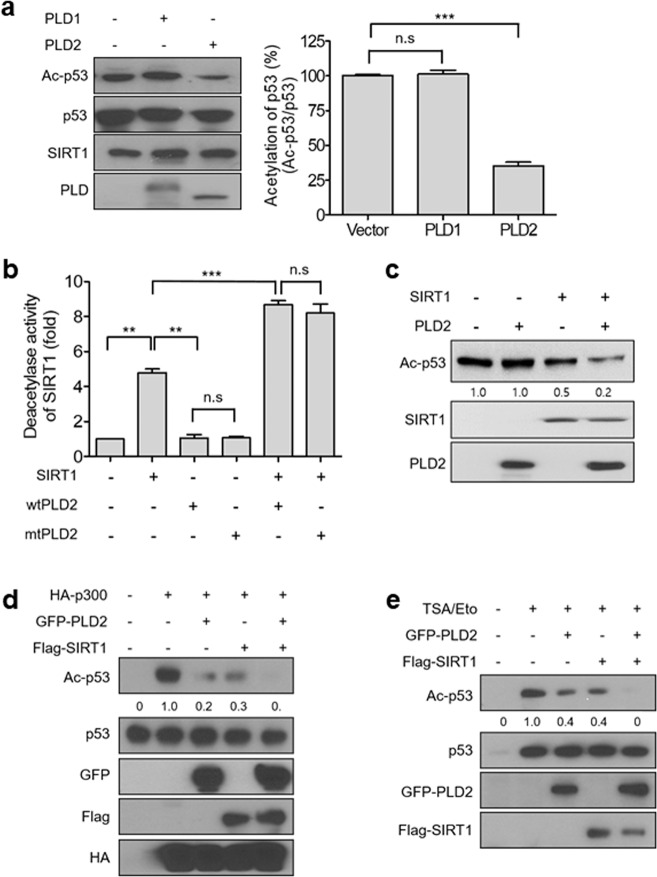
PLD2 cooperates with SIRT1 to deacetylate p53. **a** HEK293 cells were transfected with GFP-PLD1 or GFP-PLD2, and the lysates were analyzed by immunoblotting using the indicated antibodies. The intensity of the acetylated p53 bands was normalized to the intensity of p53 bands, quantified and compared. The values represent the mean ± SD of three independent experiments. ***P < 0.001, n.s., nonsignificant. **b** Effect of PLD2 on the deacetylation activity of SIRT1 in vitro. Recombinant SIRT1 was incubated with a fluorescent substrate (Lys379-382 residues of p53) in the presence of 100 μM NAD^+^. In vitro translated wt or mtPLD2 was added to the reaction mixture, and the deacetylation activity of SIRT1 was measured. The values represent the mean ± SD of three independent experiments. **P < 0.01, ***P < 0.001, n.s., nonsignificant. **c** PLD2 stimulates SIRT1-induced p53 deacetylation in vitro. In vitro acetylation of p53 was performed as described in the “Materials and methods” section. **d** p300-induced acetylation of p53 was suppressed by PLD2 in HEK293 cells transfected with the indicated constructs, and the lysates were analyzed by immunoblotting with the indicated antibodies. **e** Effect of PLD2 and/or SIRT on TSA/etoposide (Eto)-induced p53 acetylation. A549 cells were transfected with the indicated constructs and treated with 20 μM etoposide (Eto) and 500 nM TSA. Lysates were analyzed by immunoblotting using the indicated antibodies. The results are representative of at least three independent experiments.

SIRT1 is known to associate with and deacetylate p53, a transcription factor that regulates the expression of genes such as p21, BAX, and NOXA to promote cell cycle arrest, senescence, and apoptosis^11,24,25^. Ectopic expression of PLD2, but not PLD1, significantly decreased the acetylation of endogenous p53, as demonstrated by immunoblotting (Fig. 3a). Next, we examined the effect of PLD2 on the p53 deacetylase activity of SIRT1 using in vitro translated wt or catalytically inactive mutant (mt) PLD2 (K758R) and fluorescently labeled a p53 peptide (amino acids 379–382). Both wtPLD2 and mtPLD2 showed increased SIRT1-dependent p53 deacetylation activity. However, neither wt nor mutant PLD2 had an effect on p53 deacetylation in the absence of SIRT1 (Fig. 3b), indicating that PLD2 promotes SIRT1-mediated p53 deacetylation independent of its lipase activity. To rule out the possibility of an artifact caused by the fluorophore, we generated acetylated p53 protein using p300 HAT and acetyl-CoA for use in an in vitro deacetylation assay. Deacetylation was monitored by immunoblotting with an antiacetylated p53 antibody. PLD2 further stimulated SIRT1-induced p53 deacetylation but did not affect p53 deacetylation in the absence of SIRT1 (Fig. 3c). SIRT1 or PLD2 overexpression in HEK293 cells greatly suppressed the p300-induced acetylation of endogenous p53, and PLD2 and SIRT1 coexpression dramatically abolished p300-mediated p53 acetylation compared with only PLD2 or SIRT1 expression (Fig. 3d). The HDAC1 complex deacetylates p53^26^. Thus, TSA, an HDAC inhibitor, was used to maximize p53 acetylation and was added to cells along with etoposide, which causes DNA damage. In A549 cells treated with these agents, overexpression of SIRT1 or PLD2 suppressed p53 acetylation, and SIRT1 and PLD2 coexpression greatly abolished the acetylation of p53 compared with only PLD2 or SIRT1 expression (Fig. 3e). In addition, TSA/etoposide-induced p53 acetylation was suppressed by the expression of wtPLD1 and mtPLD2, suggesting PLD2 activity-independent p53 deacetylation (Supplementary Fig. 1). These results indicate that PLD2 cooperates with SIRT1 to promote p53 deacetylation.

### The binding of PLD2 with SIRT1 is required for the stimulation of SIRT1 activity

**Fig. 4:**
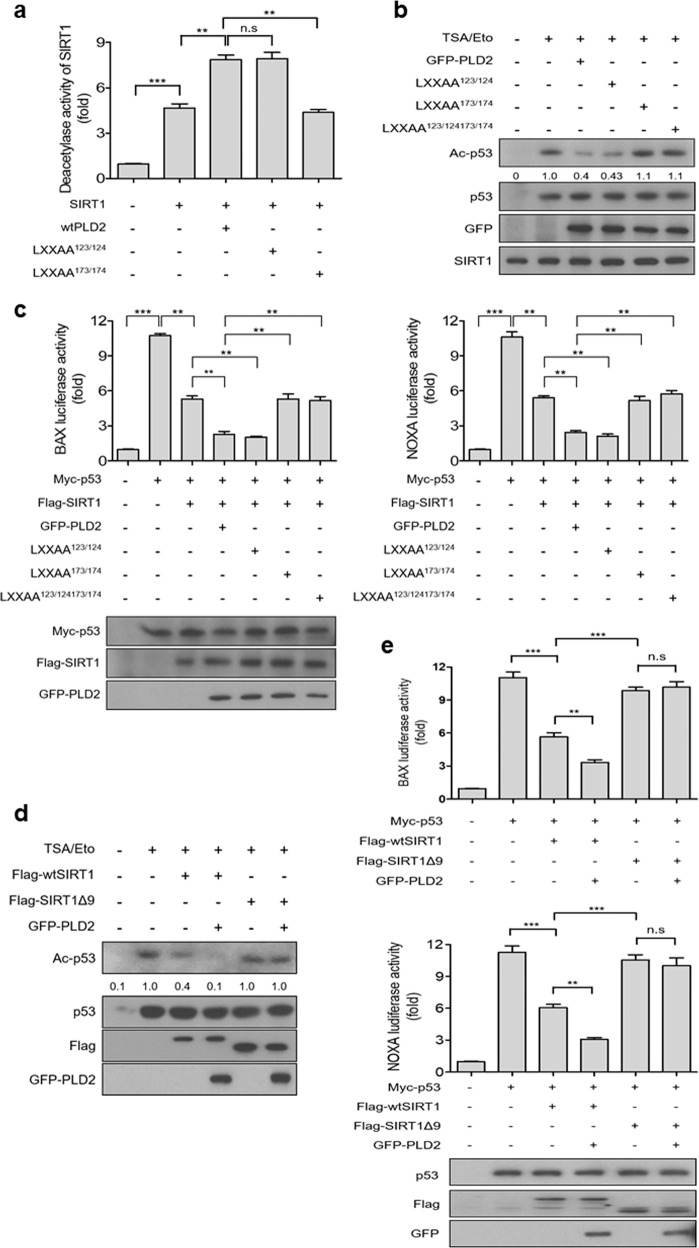
The binding of PLD2 with SIRT1 is required for the stimulation of SIRT1 activity. **a** Effect of LXXAA mutants of PLD2 on the deacetylation activity of SIRT1. Recombinant SIRT1 was incubated with a fluorescent substrate in the presence of 100 μM NAD^+^. In vitro translated wt, LXXAA^123/124^-, and LXXAA^173/174^-mutant PLD2 were added to the reaction mixture, and the deacetylation activity of SIRT1 was measured. **b** Effect of LXXAA mutants of PLD2 on p53 acetylation. A549 cells were cotransfected with the indicated constructs of PLD2 and treated with 20 μM etoposide and 500 nM TSA. Lysates were analyzed by immunoblotting using the indicated antibodies. **c** Effect of LXXLL mutants of PLD2 on the transcriptional activity of p53. HEK293 cells were cotransfected with the indicated constructs and *p53*-responsive *Bax* or *Noxa*-luciferase reporter. The luciferase activity was measured. Protein expression levels were analyzed by immunoblotting. **d** Effect of PLD2 on the deacetylation of p53 in the presence of the SIRT1 deletion mutant (SIRT1Δ9). A549 cells were transfected with the indicated constructs and treated with etoposide and TSA, followed by immunoblotting using the indicated antibodies. **e** PLD2 binding-defective SIRT1Δ9 does not cooperate with PLD2 in the transcriptional activity of p53. HEK293 cells were cotransfected with the indicated constructs and *p53*-responsive *Bax* or *Noxa*-luciferase reporter. The luciferase activity was measured. Protein expression levels were analyzed by immunoblotting. The results are representative of at least three independent experiments and are shown as the means ± SEM. **P < 0.01, ***P < 0.001, n.s., nonsignificant.

We further investigated whether the binding of PLD2 to SIRT1 is involved in the stimulation of SIRT1 activity. Because the Leu (L) residues at positions 173 and 174 of the LXXLL motif of PLD2 are required for binding with SIRT1, we performed an in vitro deacetylation assay using recombinant SIRT1 and in vitro translated PLD2 mutants. Wt and LXXAA^123/124^-mutant PLD2 significantly stimulated the deacetylase activity of SIRT1, whereas the LXXAA^173/174^-mutant PLD2 decreased SIRT1 activity compared to that of wtPLD2 (Fig. 4a). Moreover, wt and LXXAA^123/124^-mutant PLD2 attenuated TSA/etoposide-induced p53 acetylation, but mutation of residues 173 and 174 in the LXXLL motif of PLD2 did not reduce the p53 acetylation induced by TSA/etoposide (Fig. 4b). Furthermore, *Bax-* and *Noxa-*luciferase reporter assays showed that wt and LXXAA^123/124^-mutant PLD2 further promoted the SIRT1-mediated repression of p53 transcriptional activity (Fig. 4c). However, the LXXAA^173/174^ mutant of PLD2 did not affect the p53 transcriptional activity regulated by SIRT1 (Fig. 4c). We further investigated whether the SIRT1 deletion mutant inability to bind PLD2 affects p53 acetylation and transactivation. TSA/etoposide-induced p53 acetylation was greatly suppressed by wtSIRT1 but not by the SIRT1 deletion mutant lacking amino acids 610–678 (SIRT1Δ9) (Fig. 4d). Although PLD2 further promoted wtSirt1-induced p53 deacetylation, it did not affect p53 acetylation in the presence of SIRT1Δ9 (Fig. 4d). Moreover, p53-activated *Bax* and *Noxa*-luciferase reporter assays indicated that SIRT1Δ9 was defective in the suppression of p53 transactivation, and PLD2 did not inhibit p53 transactivation in the presence of SIRT1Δ9 (Fig. 4e). These results were comparable to those of p53 acetylation (Fig. 4d). Collectively, these results suggest that the binding of PLD2 to SIRT1 is required for the stimulation of SIRT1 activity.

### PLD2-induced p53 inactivation is mediated by SIRT1

**Fig. 5:**
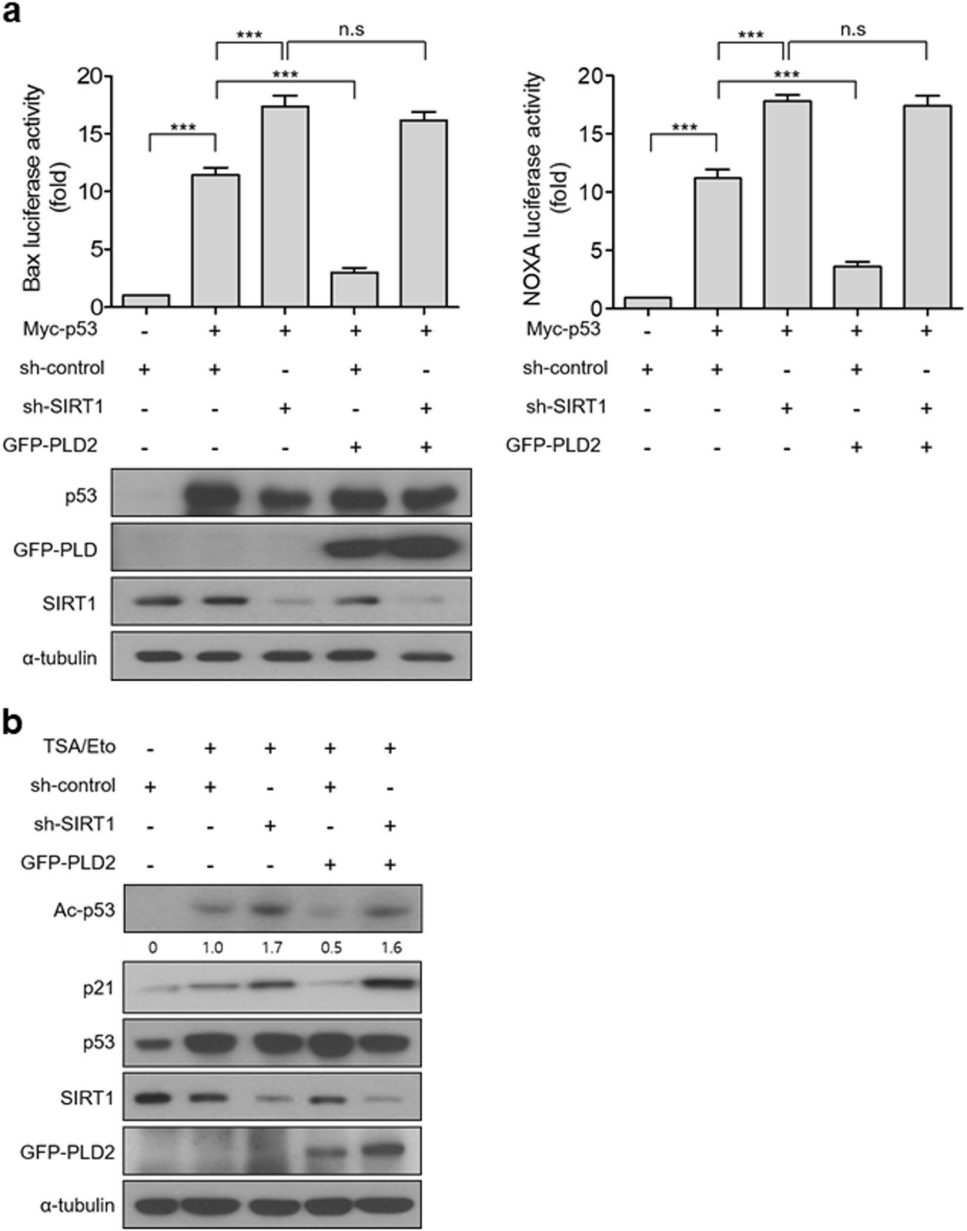
PLD2-induced p53 inactivation is mediated by SIRT1. **a** Effect of SIRT1 depletion on PLD2-induced inactivation of p53 transcriptional activity. A549 cells were transfected with the indicated constructs and *p53*-responsive *Bax* or *Noxa*-luciferase reporter. The luciferase activity was measured. Protein expression levels were analyzed by immunoblotting. **b** Effect of SIRT1 depletion on the acetylation of p53 suppressed by PLD2. A549 cells were transfected with PLD2 and shSIRT1 and treated with 30 μM etoposide and 500 nM TSA. Lysates were analyzed by immunoblotting using the indicated antibodies. The results are representative of at least three independent experiments and are shown as the mean ± SEM. **P < 0.01, ***P < 0.001, n.s., nonsignificant.

We sought to determine whether SIRT1 is required for PLD2-induced p53 inactivation. Depletion of endogenous SIRT1 significantly promoted p53-activated *Bax* or *Noxa*-luciferase reporter gene transcription compared to that of control shRNA (Fig. 5a). PLD2 significantly suppressed the p53-induced transactivation of *Bax* and *Noxa* (Fig. 5a). However, depletion of SIRT1 prevented PLD2-mediated suppression of p53 transactivation (Fig. 4a), suggesting that SIRT1 is required for PLD2-driven inhibition of p53 transactivation. The protein levels of SIRT1 and PLD2 were monitored by immunoblotting. Moreover, PLD2 or wtSIRT1 significantly suppressed p53-activated *Bax* and *Noxa*-luciferase reporter gene transcription, and coexpression of PLD2 and wtSIRT1 further suppressed the p53-mediated transactivation of *Bax* and *Noxa* (Supplementary Fig. 2). A deacetylase-defective SIRT1 mutant (H363Y) did not suppress p53-mediated transactivation. PLD2 did not affect p53-induced transactivation in the presence of a catalytically inactive SIRT1 mutant (HY) (Supplementary Fig. 2). Furthermore, we investigated whether SIRT1 affected PLD2-mediated p53 deacetylation. Treatment with TSA/etoposide-induced p53 acetylation, which was further increased by depletion of SIRT1 (Fig. 5b). The expression level of p21, a target gene of p53, was correlated with the level of p53 acetylation. SIRT1 depletion prevented PLD2-mediated p53 deacetylation (Fig. 5b). These data suggest that SIRT1 is required for PLD2-driven p53 deacetylation and suppression of p53 transactivation.

### PLD2 prevents etoposide-induced apoptosis via SIRT1

**Fig. 6:**
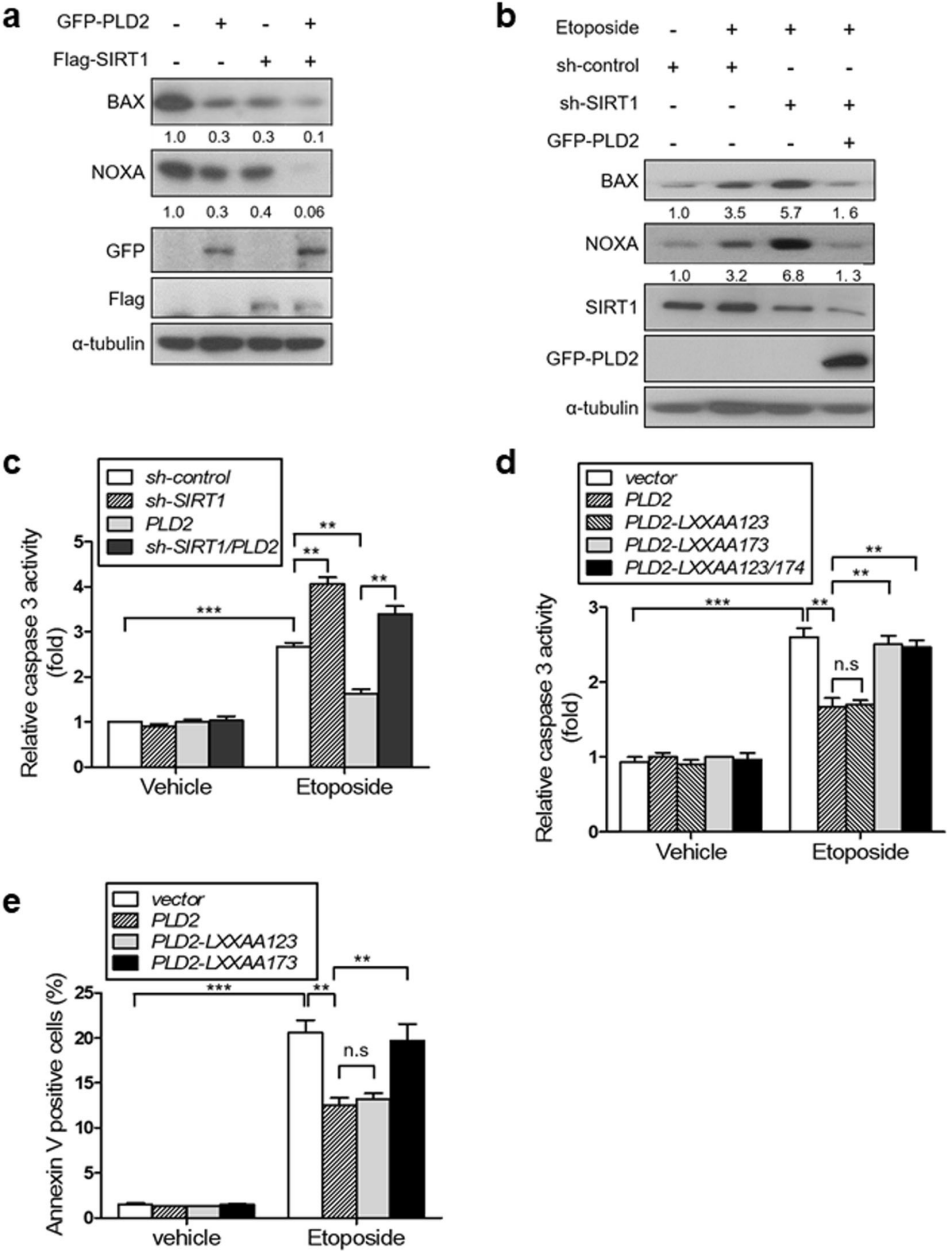
PLD2 prevents etoposide-induced apoptosis via SIRT1. **a** Effect of PLD2 and/or SIRT1 on the expression of proapoptotic genes. A549 cells were transfected with the indicated constructs, and the lysates were analyzed by immunoblotting using the indicated antibodies. The intensity of the indicated bands was normalized to the intensity of their respective α-tubulin bands, quantified, and compared. **b** Effect of PLD2 on the expression of etoposide-induced proapoptotic genes upon SIRT1 depletion. **c** A549 cells were transfected with PLD2 and/or shSIRT1 and treated with etoposide (30 μM) for 40 h, and caspase-3 activity was measured. **d** A549 cells were transfected with wt or LXXAA-mutant PLD2 and treated with etoposide. Caspase-3 activity was measured. **e** A549 cells were transfected with wt and LXXAA-mutant PLD2 and treated with etoposide for 40 h. Apoptotic cells were detected with an annexin V-FITC apoptosis detection kit. Annexin V-positive cells were then quantified. The results are representative of at least three independent experiments and are shown as the mean ± SEM. **P < 0.01, ***P < 0.001, n.s., nonsignificant.

In response to genotoxic stress, SIRT1 inhibits apoptosis and promotes cell survival through several pathways, such as p53^27–29^. To investigate whether PLD2 affects SIRT1-dependent functions, we investigated the expression of the proapoptotic target genes p53, BAX, and NOXA. Overexpression of SIRT1 or PLD2 decreased BAX and NOXA expression (Fig. 6a). Coexpression of PLD2 and SIRT1 further suppressed the expression of p53 target genes compared with the expression of either PLD2 or SIRT1 alone (Fig. 6a). We further investigated whether SIRT1 depletion affects the expression of PLD2-repressed p53 target proapoptotic genes in response to etoposide. Treatment with these agents increased BAX and NOXA expression, and this effect was further enhanced by SIRT1 knockdown (Fig. 6b). PLD2 overexpression greatly diminished the expression of p53 target proapoptotic genes induced by SIRT1 depletion. Moreover, SIRT1 depletion in A549 cells increased etoposide-induced caspase-3 activity, which was decreased by PLD2 overexpression (Fig. 6c). In addition, depletion of SIRT1 recovered etoposide-induced caspase-3 activity, which had been decreased by PLD2 overexpression. Furthermore, we investigated whether the interaction of SIRT1 with PLD2 affects the apoptosis of A549 cells. The LXXAA^173/174^ mutant of PLD2, which does not bind to or activate SIRT1, did not prevent etoposide-induced apoptosis, in contrast to wtPLD2 (Fig. 6d). Overexpression of LXXAA^123/124^-mutant PLD2 protected against etoposide-induced apoptosis to a similar extent as wtPLD2. However, the LXXAA^173/174^ mutant of PLD2 did not reduce the population of etoposide-induced apoptotic cells compared with wt or LXXAA^123/124^-mutant PLD2 (Fig. 6e). These results suggest that PLD2 may prevent etoposide-induced apoptosis via SIRT1.

## Discussion

The present study demonstrates that the LXXLL motif of PLD2 is required for SIRT1 activation and protection against etoposide-induced apoptosis via its interaction with SIRT1. PLD1 and PLD2 are overexpressed in various cancers and are intimately associated with tumorigenesis via the generation of PA^13^. Here, we show that PLD2, but not PLD1, contains the LXXLL motif in the PX domain and acts as a positive regulator of SIRT1. PLD2 mediates mitogenic signaling from growth factors and regulates receptor-induced cell survival^13,30–33^.

Although PLD-mediated physiological functions are primarily mediated by the product of its enzymatic activity, the PLD protein itself plays pivotal roles in the regulation of its biological functions through interactions with signaling biomolecules independent of its lipase activity^34^.

PLD has specific domains, such as the PX and pleckstrin homology (PH) domains, which are involved in protein–protein interactions.

The PX domain of PLD2 acts as both a GTPase-activating protein for dynamin and a potent guanine nucleotide exchange factor for many small GTPases, regardless of its other activity, and plays various roles, including a growth factor receptor in endocytosis, chemotaxis, and phagocytosis^35–37^. In addition, the PLD2-PX domain directly binds to and activates PKCζ, which is critical for the survival of cancer cells^38^. The PH domains of PLD1 and PLD2 have been implicated as modulators of the membrane recycling machinery that results in regulated growth factor receptor endocytosis and are linked to protein–protein interactions in cellular signaling^39,40^. Thus, PLD plays various physiological roles in a lipase activity-dependent and lipase activity-independent manner.

While the enzymatic activity of PLD isozymes is critical for the increased level of HIF-1α via promotion of translation, the PLD protein itself destabilizes the HIF-1α protein by interacting directly with the components involved in von Hippel–Lindau-dependent degradation of HIF-1α, independent of PLD activity. The PH domain of PLD isozymes interacting with these proteins promoted degradation of HIF-1α independent of oxygen concentration and suppressed tumor progression^41,42^. Moreover, the PH domain of PLD2 acts as a negative regulator of focal adhesion kinase^43^. Thus, the PH domain of PLD isozymes might be useful in the development of therapeutics targeting HIF-1α and FAK in cancers.

The LXXLL motifs play a pivotal role in protein–protein interactions associated with various transcriptional regulations, and the majority of these LXXLL-mediated interactions involve associations between transcription factors and coactivators^44^. The LXXLL motif was originally identified as a cofactor that binds to hormone-activated nuclear receptors^20^. Functionally active LXXLL motifs have also been documented in proteins that do not directly interact with nuclear receptors, including several transcription factors^6,45^. These motifs mediate interactions that can activate or repress transcription. Nonfunctional LXXLL motifs can also be found in proteins involved in transcriptional regulation or other cellular processes.

However, the biological roles of SIRT1 are mostly revealed through its deacetylation of nonhistone substrates, which have been discovered in increasing numbers. These nonhistone substrates are involved in a wide variety of cellular functions, particularly in metabolic, oxidative/genotoxic, and oncogenic stress responses.

Thus, it is likely that the discovery of the LXXLL motif fails does not encompass the complexity of the system. Many factors, including availability of proteins for interaction and the composition of flanking residues, might contribute to determining the binding affinity and enabling discrimination between potential binding partners. Thus, further characterization of LXXLL motif interactions is required to understand the role of flanking sequences in determining binding specificities.

LXXLL^173/174^ with leucine at position 173/174 in PLD2, but not LXXLL^123/124^, is critical for PLD2 interaction with and activation of SIRT1. Moreover, it has been suggested that proteins that interact with LXXLL motifs necessarily undergo structural changes, thus providing a mechanism for modulating binding^44^. Thus, it has been speculated that the binding of LXXLL^173/174^ of PLD2 with the C-terminal domain of SIRT1, which is essential for deacetylase activity, might induce conformational changes that stimulate SIRT1 activity. Although the function of LXXLL^123/124^ remains unknown, it is possible that LXXLL^123/124^ might play a differentiating role via interaction with other binding partners.

SIRT1 plays crucial roles in many cellular events, including aging, cancer, and cellular reprogramming. In response to cellular stress, including DNA damage, hypoxia, and reactive oxygen species production, nuclear p53 is acetylated, leading to its stabilization and increased DNA-binding activity. This activity further increases the transcription of genes in the cascades of antioxidants, oxidants, and/or apoptosis. SIRT1 deacetylates p53 and suppresses p53-mediated transcriptional activity, thereby reducing the expression of its downstream molecules involved in cell cycle arrest and apoptosis. Thus, SIRT1 can promote tumor growth by increasing the rates of apoptosis through the suppression of p53. It has been suggested that SIRT1 is essential for cell survival because it inactivates p53-induced apoptosis^46^.

Since the expression of PLD2 and SIRT1 is upregulated in various cancers, the interaction between proteins may be increased in cancers and contribute to antiapoptotic function.

However, SIRT1 may reportedly act as a tumor suppressor in certain cellular conditions, suggesting its contradictory role in cancer development and progression^47^. Thus, it remains controversial whether SIRT1 acts as a tumor promoter or suppressor. Elucidation of the regulatory mechanism of the SIRT1-p53 axis might provide insights into controlling the signaling pathways involved in the development of potential therapeutic interventions for treating cancer. Although many SIRT1 substrates have been documented, relatively little is known about the regulators of SIRT1 activity. Therefore, the source of SIRT1 regulation is of considerable interest. Here, we show that PLD2 directly regulates SIRT1 expression to suppress p53 acetylation and transcriptional activation, thereby protecting against DNA damage-induced apoptosis. The interaction between PLD2 and Sirt1 is required for protection of cancer cells from apoptosis. Blocking the interaction between the proteins may promote apoptosis of cancer cells. For a potential therapeutic approach, modified LXXLL-containing peptides in PLD2 might suppress the interactions between the LXXLL motif and SIRT1; thus, these findings not only provide insights into the functional importance of regulatory interactions involving this motif but may also represent promising therapeutic strategies for ameliorating DNA damage-induced apoptosis.

## Supplementary information

Supplementary Information
