# Supplementary material for: Phospholipase D2 is a positive regulator of sirtuin 1 and modulates p53-mediated apoptosis via sirtuin 1
Source: Exp Mol Med. 2021 Sep 1;53(9):1287–97. doi: 10.1038/s12276-021-00659-y (PMC8492672; doi:10.1038/s12276-021-00659-y)

**Supplementary Information**

**Supplementary Figure legends**

**Supplementary Figure 1** Effect of wt or mtPLD2 on TSA/etoposide (Eto)-induced p53 acetylation. A549 cells were transfected with the indicated constructs and treated with 20 μM etoposide (Eto) and 500 nM TSA. The lysates were analyzed by immunoblotting using the indicated antibodies. Results are representative of at least three independent experiments.

**Supplementary Figure 2** Effect of a catalytically inactive SIRT1 mutant on PLD2-induced inactivation of p53 transcriptional activity. A549 cells were transfected with the indicated constructs and *p53*-responsive Bax or *Noxa* luciferase reporter. The luciferase activity was measured. The expression levels of the proteins were analyzed by immunoblotting. Results are representative of at least three independent experiments and shown as the mean ± SEM. **P < 0.01, n.s., nonsignificant.

**Supplementary Figures**

**Supplementary Figure 1**


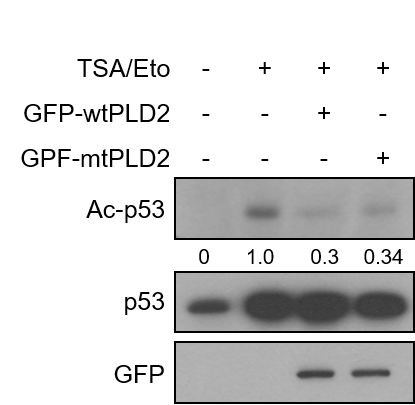


**Supplementary Figure 2**


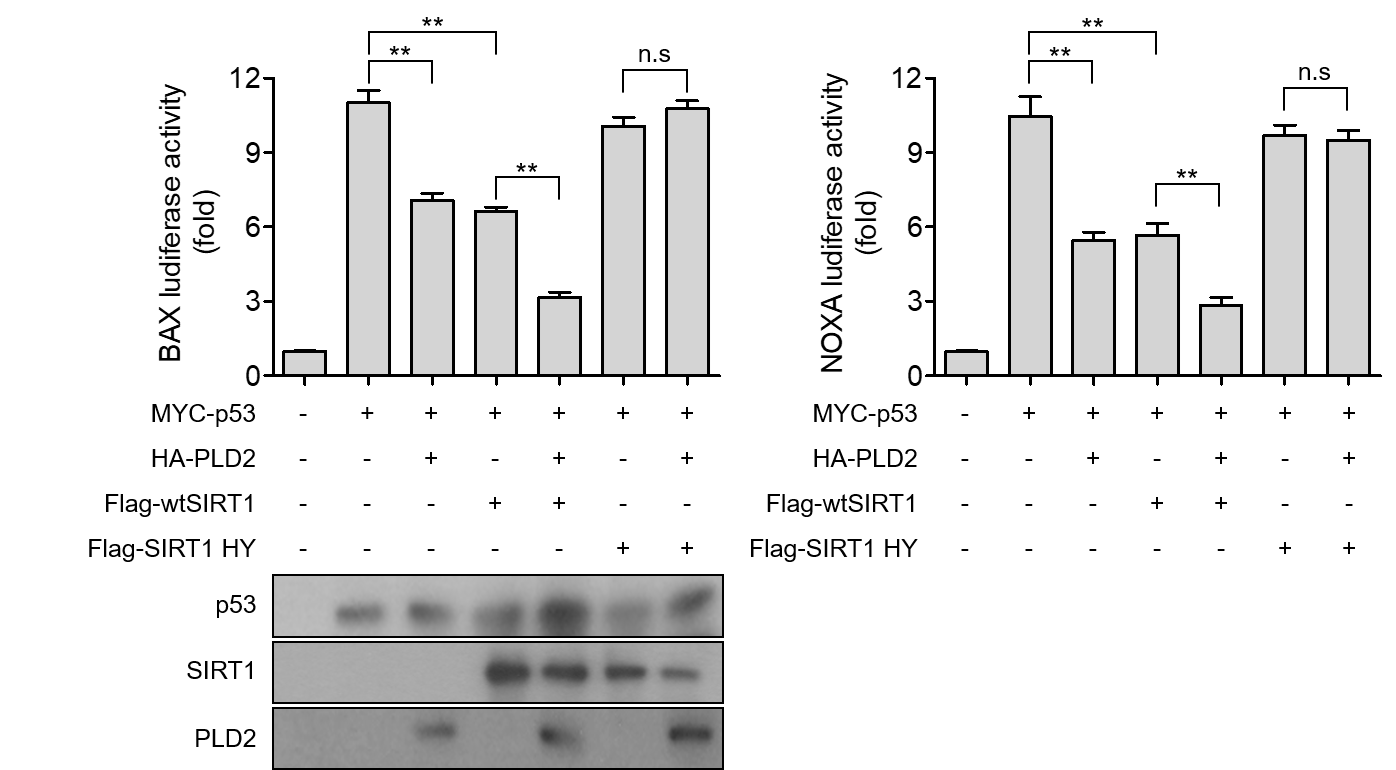

Supplement: Supplementary file 1 — Supplementary Information [file 12276_2021_659_MOESM1_ESM.docx]
